# When the Loss Costs Too Much: A Systematic Review and Meta-Analysis of Sarcopenia in Head and Neck Cancer

**DOI:** 10.3389/fonc.2019.01561

**Published:** 2020-02-05

**Authors:** Xin Hua, Shan Liu, Jun-Fang Liao, Wen Wen, Zhi-Qing Long, Zi-Jian Lu, Ling Guo, Huan-Xin Lin

**Affiliations:** ^1^State Key Laboratory of Oncology in South China, Collaborative Innovation Center for Cancer Medicine, SunYat-sen University Cancer Center, Guangzhou, China; ^2^Department of Radiotherapy, Sun Yat-sen University Cancer Center, Guangzhou, China; ^3^Department of Radiation Oncology, Sichuan Cancer Hospital & Institute, Sichuan Cancer Center, School of Medicine, University of Electronic Science and Technology of China, Chengdu, China; ^4^Department of Radiation Oncology, National Cancer Center/National Clinical Cancer Research Center for Cancer/Cancer Hospital & Shenzhen Hospital, Chinese Academy of Medical Sciences and Peking Union Medical College, Shenzhen, China; ^5^Guangdong Key Laboratory of Nasopharyngeal Carcinoma Diagnosis and Therapy, Department of Nasopharyngeal Carcinoma, Sun Yat-sen University Cancer Center, Guangzhou, China

**Keywords:** head and neck cancer, sarcopenia, meta-analysis, prognostic factor, skeletal muscle mass (SMM)

## Abstract

**Purpose:** Whether or not skeletal muscle mass (SMM) depletion, known as sarcopenia, has significant negative effects on the prognosis of patients with head and neck cancer (HNC) is both new and controversial. In this meta-analysis, we aimed to determine the prognostic significance of sarcopenia in HNC.

**Methods:** We searched PubMed, the Cochrane Library, Embase, and Web of Science, which contain trial registries and meeting proceedings, to identify related published or unpublished studies. We used the Newcastle–Ottawa Scale (NOS) to appraise the risk of bias of the included retrospective studies. Pooled hazard ratios (HR) and the *I*^2^ statistic were estimated for the impact of sarcopenia on overall survival (OS) and relapse-free survival (RFS).

**Results:** We analyzed data from 11 studies involving 2,483 patients (39.4% on average of whom had sarcopenia). Based on the univariate analysis data, the sarcopenia group had significantly poorer OS compared to the non-sarcopenia group [HR = 1.97, 95% confidence interval (CI): 1.71–2.26, *I*^2^ = 0%]. In the cutoff value subgroup, group 1, defined as skeletal muscle index (SMI) of 38.5 cm^2^/m^2^ for women and 52.4 cm^2^/m^2^ for men (HR = 2.41, 95% CI: 1.72–3.38, *I*^2^ = 0%), had much poorer OS. In the race subgroup, the results were consistent between the Asia (HR = 2.11, 95% CI: 1.59–2.81) and non-Asia group (HR = 1.92, 95% CI: 1.64–2.25). The sarcopenia group also had significantly poorer RFS (HR = 1.74, 95% CI: 1.43–2.12, *I*^2^ = 0%).

**Conclusions:** Presence of pre-treatment sarcopenia has a significant negative impact on OS and RFS in HNC compared with its absence. Further well-conducted studies with detailed stratification are needed to complement our findings.

## Introduction

Head and neck cancer (HNC) is a complex heterogeneous disease; numerous covariates affect its survival outcomes. According to National Comprehensive Cancer Network (NCCN) guidelines, radiotherapy (RT) with or without chemotherapy is the main treatment method for locally advanced HNC (1). Due to the local toxic effects of RT and chemoradiotherapy, patients with HNC may experience significant progressive weight loss and muscle mass depletion, which eventually lead to poor prognosis (2–4). Although weight loss is commonly used in clinical settings to screen for the risk of adverse outcomes in HNC, there are no universally recognized clear and reliable conclusions on the association of skeletal muscle mass (SMM) depletion and prognosis in HNC.

The main factors affecting treatment outcome are tumor characteristics and host-related factors (including age, sex, and nutritional status). Patients with HNC have a much higher risk of malnutrition than patients with other malignancies (5). Cancer patients with malnutrition typically lose lean body mass and muscle mass, while fat mass may remain or even increase (6). Muscle mass depletion, known as sarcopenia, can theoretically affect the treatment tolerance and prognosis of patients with HNC.

Sarcopenia is officially defined as generalized and progressive low SMM and function, and is related to physical disability and functional impairment (7). Sarcopenia in HNC can be quantified by the cross-sectional area in square centimeters (cm^2^) divided by the squared height in meters (m^2^) at the third lumbar (L3) or cervical (C3) vertebra level using computed tomography (CT) imaging (8). Recent studies have shown that sarcopenia is associated with increased risk of complications after tumor therapy and reduced disease-free survival (DFS) and overall survival (OS) (9–12). Sarcopenia and its effect on treatment-related complications and the clinical prognosis of HNC have recently attracted research attention. However, underestimation of the importance of sarcopenia continues to evolve when compared to the large number of studies that have been focused on different patient- and disease-related variables affecting the prognosis of patients with HNC (13–16).

Currently, whether sarcopenia in HNC can act as a prognostic factor is both little well-known and controversial (17, 18). Accordingly, we conducted this meta-analysis to investigate the prevalence of sarcopenia in patients with HNC and to determine its impact on clinical prognosis.

## Methods

### Search Strategy

The prospective registration number of this meta-analysis on PROSPERO was CRD42019128406. This study was approved by the Ethics Committee of Sun Yat-sen University Cancer Center. Databases such as PubMed, the Cochrane Library, Embase, and Web of Science, which contain trial registries and meeting proceedings, were searched before August 30, 2019. In each database, we used the same search term: (“sarcopenia” or “fragility” or “sarcopenic” or “muscle index” or “muscle mass” or “muscle depletion” or “muscular atrophy”) and (“head and neck cancer” or “head and neck neoplasm” or “HNSCC”). The language restriction was English; there were no other filters.

### Study Selection

At the full-text screening step, two reviewers (X.H. and S.L.) assessed the relevant literature independently for inclusion. The κ statistic was used for inter-rater reliability (19). The inclusion criteria were as follows: (1) cohort and case–control study; (2) studied patients with HNC(s); (3) reported SMM or function measurement; and (4) reported prognostic data such as OS, progression-free survival (PFS), or DFS. Studies were excluded if data on the impact of sarcopenia on survival outcomes were unavailable.

### Data Extraction

The two reviewers (S.L. and X.H.) extracted data from primary texts and Supplementary Appendixes independently and summarized them in a standardized data abstraction form. The extracted items are partly listed in Table 1. The results were reconciled and a third reviewer (J.F.L.) was consulted if there were discrepancies. In the case of missing data, the authors of the study in question were contacted via e-mail. If the authors did not reply, data from the published articles were used.

**Table 1:** Characteristics of included studies.

**Author year**	**Country**	**Cancer**	**Stage**	**No. of patients**	**Age**	**Follow-up** **(months)**	**Sarcopenia** **assessment**	**Cut point (cm^2^/m^2^)**			**Sarcopenia** **(%)**	**Treatment**	**Outcome**	**Adjusted major confounders**	**NOS score**
								**Female**	**Male**	**Methods**					
Ganju et al. (20)	America	Head and neck excluding p16+ oropharynx cancer	AJCC 7^th^ III–IVB	246	60 (19–88)	35.1 (1–83)	^*^L3 SMI	41	43 or 53 by BMI	Martin et al. (12)	58	CCRT/IC+CCRT, Surgery+	OS PFS	Baseline BMI, Age, Sex, Race, Site, Stage, Smoke, Treatment	**7**
Stone et al. (21)	America	Head and neck	AJCC I–IVB	260	61.1 (±11)	ND	L3 SMI	38.5	52.4	Prado et al. (6)	55.4	Surgery ± RT/CRT	OS	Baseline BMI, Stage, Smoke, ALB, HPV, Treatment	**7**
Bril et al. (22)	Netherlands	Larynx and Hypopharynx	AJCC 6/7^th^ 0–IV	235	64.7 (±9.1)	62.4	^*^L3 SMI	43.2	43.2	Wendrich et al. (23)	46.4	Surgery ± pre Chemo/RT ± adjuvant treatment	OS	Baseline BMI, Sex, Smoke, Site, Treatment	**7**
Jung et al. (24)	Korea	Head and neck	AJCC 7^th^ III–IV	258	64 (56–73)	53.6 (26.3–70.5)	L3 SMI	38.5	52.4	Prado et al. (6), Mourtzakis et al. (25)	6.6	Surgery ± RT/CCRT	OS DFS	Baseline Age, CCI, ALB, Site, HPV-P16, Smoke, Treatment	**7**
^†^Van Rijn–Dekker et al. (26)	Netherlands	HNSCC	AJCC I–IVB	750	ND	ND	^*^L3 SMI	30.6	42.4	Lowest gender-specific quartile	25	Chemo/RT	OS DFS	Baseline Age, WHO score, stage, site	**6**
Cho et al. (17)	Korea	Head and neck	AJCC III–IVB	221	59 (18–94)	30 (1–110)	L3 SMI	31	49	Go et al. (27), Kim et al. (28)	48.0	RT/ CCRT/ IC+CCRT	OS, PFS	Univariate analysis	**7**
^‡^Fattouh et al. (29)	America	HNSCC	AJCC 6/7^th^ M0	113	ND	≥60	L3 SMI	38.5	52.4	Prado et al. (30), Mokdad et al. (31)	64.6	Chemo/RT, Surgery+	OS	Baseline BMI, Age, Sex, Stage, Treatment	**8**
Grossberg et al. (18)	ND	HNSCC	AJCC 7^th^ M0	190	57.7 (±9.4)	68.6	L3 SMI	38.5	52.4	Prado et al. (6), Parsons et al. (32)	35.3	RT/CCRT/IC+CCRT, Surgery+	OS,	Baseline BMI, Age, Sex, Smoke, Site, Stage, Treatment, HIV, Diabetes, Cardiovascular disease	**8**
Nishikawa et al. (33)	Japan	HNSCC	M0	85	66 (28–89)	29.6 (1–40.7)	L3 SMI	30.3	46.7	Prado et al. (6)	46.0	RT/ CCRT/ BioRT/Surgery, NACT+	OS	Baseline weight loss, ALB, CRP	**6**
Tamaki et al. (34)	Japan	SCC of oropharyngeal	AJCC II–IVC	113	Non-sarcopenia 57.63 (±10.25); sarcopenia 63.5 (±12.91)	0–120	L3 SMI	41	41or 43	Martin et al. (12)	28.3	CCRT/surgery ± adjuvant treatment	OS DFS	Baseline BMI, HPV-P16, Sex, Smoke, Alcohol	**6**
Wendrich et al. (23)	Dutch	HNSCC	AJCC III-IV (locally advanced)	112	54.5 (±9.4)	15–90	^*^L3 SMI	43.2	43.2	Non-gender-specific optimal stratification	54.5	CCRT	OS	Univariate analysis	**6**

* *L3 SMI was calculated by C3 SMI using the method from Swartz et al. (8)*.

† *Research as a conference meeting paper and the author provided information about sarcopenia (%)*.

‡ *Research does not have a univariate analyzed OS data*.

AJCC, American Joint Committee on Cancer; BMI, body mass index; HNSCC, head and neck squamous cell carcinoma; CCRT, concurrent chemoradiotherapy; IC, induction chemotherapy; L3, the third lumbar vertebra; No., number; ND, no description; OS, overall survival; DFS, disease-free survival; DSS, disease-specific survival; M, metastasis; NOS, Newcastle–Ottawa Scale; PFS, progression-free survival; RT, radiation therapy; SMI, skeletal muscle index.

*Bold represents the value of NOS-Score*.

### Risk of Bias Assessment

Two reviewers (W.W. and Z.Q.L.) assessed the bias independently. We used the modified Newcastle–Ottawa Scale (NOS) (35), which involves patient selection, study group comparability, and assessment of outcomes, to appraise the methodological quality of the included retrospective studies. The quality of each cohort study was scored 0–9, and case–control studies were scored 0–10; studies with scores of at least 6 were deemed good quality (19).

### Meta-Analysis

We calculated and subsequently pooled in standard meta-analyses and hazard ratios (HRs) with corresponding 95% confidence intervals (95% CIs) for survival outcomes. HR and its 95% CI were directly used if these values were reported; otherwise, the natural logarithm of the HR (lnHR) and standard error of the lnHR [se(lnHR)] were calculated to determine the pooled HRs and 95% CIs according to the method of Parmar et al. (36) and Tierney et al. (37). The χ^2^ and *I*^2^ tests were used to appraise statistical heterogeneity between studies, with significance set at *P* < 0.10. The random-effects model was consistently used to maintain a conservative conclusion. Exploratory subgroup analyses were also performed. Potential publication bias was quantitatively assessed by funnel plot and quantified by the Egger test (38) and the trim-and-fill method (39) using Stata 14.0 (Stata Corp, College Station, TX, USA). The meta-analyses were performed using Review Manager 5.3 (Cochrane Collaboration, Oxford, UK).

## Results

### Search Strategy

**Figure 1:**
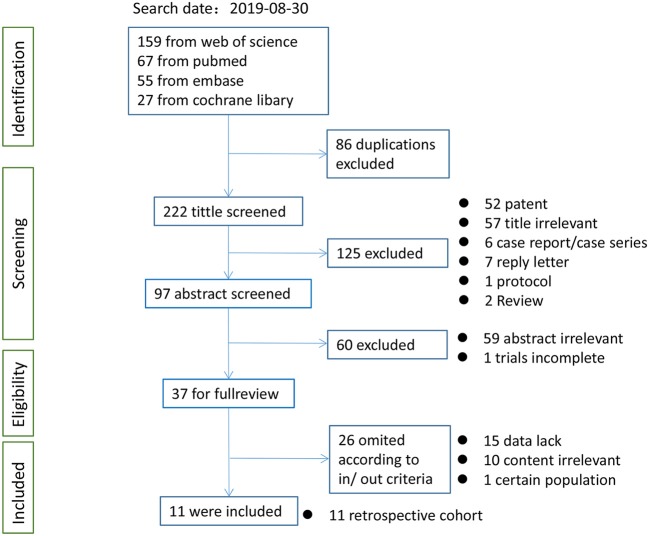
Flow chart of study selection for meta-analysis.

After the initial literature search on August 30, 2019, 11 studies (17, 18, 20–24, 26, 29, 33, 34), including one nested case–control study (29) and a meeting abstract (26), assessing 2,483 patients were pooled in the present meta-analysis. Of the patients involved, an average of 39.4% had sarcopenia (979 patients and 1,504 patients had and did not have sarcopenia, respectively, according to different cutoff values; Figure 1). The kappa coefficient was 0.842 (Figure S1).

### Characteristics of the Studies

Table 1 summarizes the characteristics of the 11 retrospective studies. Four studies (17, 24, 33, 34) were from Asia, i.e., Japan and Korea. All studies included patients with non-metastatic clinical stage, except the cohort of Tamaki et al. (34), which included four patients with stage IVC disease. All studies used the SMI, quantified by the cross-sectional area in cm^2^ divided by m^2^ at the L3 or the C3, and then calculated the L3 vertebra level mainly using CT imaging. There were different sarcopenia cutoff definitions (6, 12, 25, 27, 28, 30–32); three studies (22, 23, 26) used self-defined definitions to obtain optimum stratification. Sarcopenia prevalence ranged from 6.6 to 64.6%. The HRs from nine studies were adjusted for major confounders such as baseline body mass index (BMI) etc. The quality of all included studies was fair (Table S1). All studies had low risk of bias, with NOS scores of 6–8. HR and 95% CI data from two studies (17, 23) were extracted and estimated from survival curves using indirect methods. Lastly, no authors except Van Rijn-Dekker (26) replied to our query e-mails; therefore, we used only the available published data.

### Overall Survival

**Figure 2:**
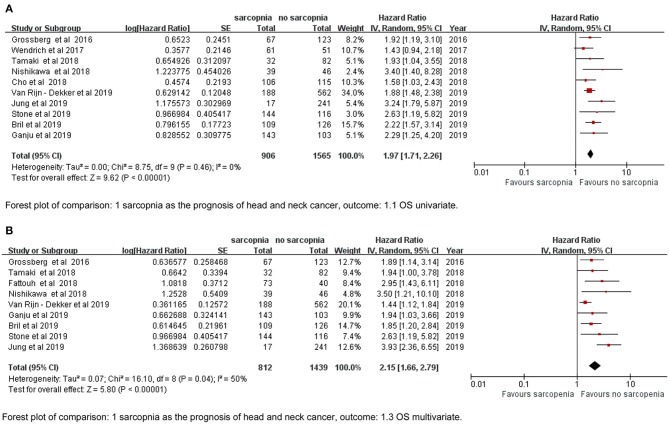
Forest plot of **(A)** univariate data of OS, **(B)** multivariate data of OS.

The meta-analysis of the univariate and multivariate data of the influence of the SMI on OS using the random-effects model is depicted in (Figures 2A,B). The sarcopenia group had significantly poorer OS compared to the non-sarcopenia group (in Figure 2A HR = 1.97, 95% CI: 1.71–2.26, *I*^2^ = 0% and *P* = 0.46; in Figure 2B HR = 2.15, 95% CI: 1.66–2.79, *I*^2^ = 50% and *P* = 0.04). Table 2 shows the exploratory subgroup analyses. In the primary SMI subgroup, the L3 SMI calculated from the C3 SMI showed results consistent with the L3 primary SMI (HR = 1.90, 95% CI: 1.60–2.25; HR = 2.12, 95% CI: 1.66–2.71, respectively). In the three subgroups according to cutoff values, group 1, defined as SMI of 38.5 cm^2^/m^2^ for women and 52.4 cm^2^/m^2^ for men, had much poorer OS (HR = 2.41, 95% CI: 1.72–3.38, *I*^2^ = 0%). Sarcopenia had a similar impact on the Asia and non-Asia subgroups (HR = 2.11, 95% CI: 1.59–2.81; HR = 1.92, 95% CI: 1.64–2.25, respectively). There was no difference between the high-quality group with NOS ≥ 7 and intermediate-quality group with NOS = 6 (HR = 2.13, 95% CI: 1.74–2.60; HR = 1.83, 95% CI: 1.48–2.26, respectively). As the χ^2^ test *P*-value of 0.46 and an *I*^2^ of 0% indicated consistency between the studies (Figure 2A), we did not perform sensitivity analysis except for multivariate meta-analysis for OS (Table S2).

**Table 2:** Subgroup analyses of the prognostic effect on OS of the sarcopenia vs. non-sarcopenia group in head and neck cancer.

**Variable**	**Subgroups**	**Availability**		**Effect**		**Heterogeneity**	
		**Studies** **(*N*)**	**Patients** **(*N*)**	**HR** **(95% CI)**	***P*-values**	***I*^**2**^ (%)**	**$p_{inter}^{*}$**
Race	Asian Non-Asian	4 6	678 1,793	2.11 [1.59, 2.81] 1.92 [1.64, 2.25]	<0.00001 <0.0001	39 0	0.5
Stage	Locally advanced	4	837	1.92 [1.35, 2.73]	0.0003	49	0.95
	Non-metastasis	5	1,520	2.04 [1.71, 2.42]	<0.0001	0	
	Contained M1	1	114	1.94 [1.04, 3.55]	0.04	–	
Primary SMI	L3	6	1,128	2.12 [1.66, 2.71]	<0.00001	7	0.47
	C3	4	1,343	1.90 [1.60, 2.25]	<0.00001	0	
^†^Cutoff	Group1	3	708	2.41 [1.72, 3.38]	<0.0001	0	0.45
	Group2	3	1,056	1.87 [1.47, 2.38]	<0.00001	14	
	Group3	4	707	1.92 [1.53, 2.41]	<0.00001	0	
NOS quality	NOS ≥ 7 NOS = 6	6 4	1,410 1,061	2.13 [1.74, 2.60] 1.83 [1.48, 2.26]	<0.0001 <0.00001	0 8	0.3
HR data extract	Directly Indirectly	8 2	2,138 333	2.11 [1.81, 2.47] 1.50 [1.11, 2.03]	<0.00001 0.008	0 0	0.05

^*^P_inter_ represents the significance of heterogeneity between subgroups calculated by Revman software.

† *Cutoff value in Group 1: 38.5 cm^2^/m^2^ for women and 52.4 cm^2^/m^2^ for men; Group 2: 30.3–31 cm^2^/m^2^ for women and 42.4–49 cm^2^/m^2^ for men; Group 3: 41–43.2 cm^2^/m^2^ for women and 41–43.2 cm^2^/m^2^ for men*.

*N, number; HR, hazards ratio; NOS, Newcastle–Ottawa Scale*.

### Relapse-Free Survival

**Figure 3:**
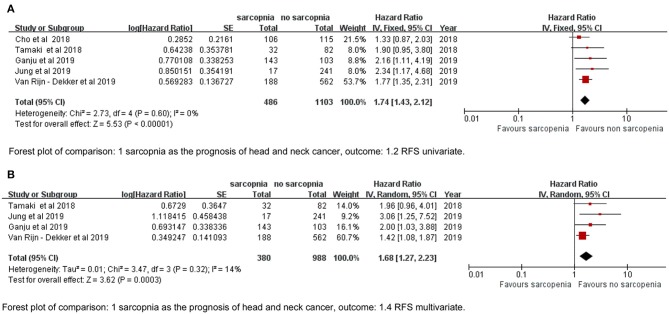
Forest plot of **(A)** univariate data of RFS, **(B)** multivariate data of RFS.

We defined RFS as the interval between diagnosis to the detection of first progression, death from any cause, or last follow-up that represented PFS in the study by Cho et al. (17) and DFS by Tamaki et al. (34). The sarcopenia group had significantly poorer RFS based on both univariate and multivariate data (HR = 1.74, 95% CI: 1.43–2.12, *P* < 0.00001, *I*^2^ = 0%; HR = 1.68, 95% CI: 1.27–2.23, *P* = 0.003, *I*^2^ = 14%; Figures 3A,B).

### Publication Bias

The publication bias test results are not separately reported (Figures S2, S3). In accordance with the funnel plot in Figure S3, Egger's test indicated a high likelihood of reporting bias (*P* = 0.035); however, the trim-and-fill method indicated that three hypothetical studies were filled in while the final conclusion remained unchanged (Figure S2).

## Discussion

Sarcopenia, known as the loss of SMM and function, is common in patients with various solid cancers with incidence ranging from 11 to 74% (40, 41). Following digestive cancer, patients with HNCs have a higher risk of experiencing malnutrition than patients with other cancer types (5, 42), due to the impact of the special tumor location and more serious treatment toxicity on the food intake. Accordingly, several recent studies have further explored the predictive value of sarcopenia in treatment-related complications and the prognosis of survival in HNC. Wendrich et al. (23) found that sarcopenia increased the risk of chemotherapy dose-limiting toxicity (CDLT) in patients with LA-HNSCC receiving chemoradiotherapy (44.3 vs. 13.7%, *P* < 0.001). Achim et al. (43) showed that up to 77% of patients with laryngeal cancer had preoperative sarcopenia and that sarcopenia was an independent predictor for all complications of total laryngectomy. Wendrich et al. (23) did not find a significant OS reduction for low SMM (*P* = 0.187). Grossberg et al. (18) found that, in patients with HNSCC, pre-RT SM depletion was no longer prognostic when BMI was included in the multivariate analysis. Indeed, obese patients without sarcopenia have significantly better prognosis than obese patients with sarcopenia (sarcopenia obesity) (6, 44). Therefore, as a nutrition-related indicator, whether sarcopenia independently affects the prognosis of HNC is appealing.

This is the first meta-analysis to report quantitative assessment of SMI and prognosis in HNC. The pooled HRs show that pre-treatment sarcopenia is significantly associated with poorer OS and RFS. The univariate HRs for survival outcomes were used to derive conclusions because we believed and observed that the multivariate meta-analysis that negative results did not participate in could be a source of publication bias.

We found relatively significant heterogeneity (*I*^2^ = 50%) in the multivariate meta-analysis for OS (Figure 2B). It appears that results by Jung et al. (24) and Van Rijn-Dekker et al. (26) are debatable (Table S2). The former had a much higher risk than any other research (HR = 3.93, 95% CI; 2.36–6.55). Interestingly, the cutoff value they used was the same as that from three other included articles (18, 21, 29) (Table 1), but the incidence rate of sarcopenia was only 6.6%; the possible reasons for this are as follows: (a) 6.6% is for sarcopenia with visceral obesity in their study, (b) the locally advanced cancer stage is the distinguishing property, or (c) there might be potential bias that affected the incidence. The study by Van Rijn-Dekker et al., which will soon be published in full, was a meeting abstract that investigated a large-scale cohort of 750 patients with HNSCC, the incidence of sarcopenia was also low, i.e., as 25%, and the result was conservative and narrow (HR = 1.44, 95% CI: 1.12–1.84). In their e-mail reply, the cutoff was set by the lowest sex-specific quartile categorized in our Group 2 cutoff subgroup. Group 2 was less good enough to report a prognostic effect of sarcopenia than Group 1, which is based on log-rank statistics to separate patients with sarcopenia (6) (Table 2), so we agree that setting a cutoff for sarcopenia by using the log-rank test may be better. It is common to obtain the head and neck CT in HNC, and we also did not observe significant intergroup heterogeneity between the primary site of SMI definition subgroups (C3 or L3) (Table 2). We suggest that more studies should explore the effect and cutoff value of neck muscles on HNC prognosis.

In our review, sarcopenia had a similar impact on the Asia and non-Asia subgroups, which suggest that sarcopenia could be widely used. Sarcopenia was not a prognostic factor for p16+ oropharyngeal cancer (34, 45), and maybe different tumor types that caused a wide range of prognosis have specific influence on sarcopenia; thus, it is imperative for further studies on particular and rare types of tumors other than p16+ oropharyngeal cancer to determine the prognostic value of sarcopenia. As for the set of cutoff value, the low intergroup heterogeneity indicates that different cutoffs could all be used (Table 2). Therefore, a unitary cutoff is not reasonable, and it can be inferred that using different races, tumor-node-metastasis (TNM) clinical stages, tumor types, age groups, and other features to form the appropriate multi-factor model can identify patients with poor prognosis as accurately as possible.

Our study also aims to turn its attention to the routine evaluation and intervention of sarcopenia for HNC. Many strategies can be attempted to prevent and treat sarcopenia. Among them, lifestyle modification, specific dietary habits, and therapeutic measures have been recommended. Protein supplementation and regular resistance exercise are the mainstream treatments of sarcopenia: to increase muscle mass and help augment muscle strength (46, 47). In addition, drugs that can block the cytokines associated with the muscle atrophy signaling pathways [such as myostatin/activin, interleukin (IL)-6, and tumor necrosis factor (TNF)-α] or medications that induce signals of muscle hypertrophy (such as growth hormone agonists, ghrelin, and anabolic steroids) may be useful for sarcopenia accompanied by visceral obesity (48).

Due to the retrospective nature of the included studies, the present meta-analysis has several limitations. First, only some of those articles included the treatment variable, which is a significant prognostic factor for survival outcomes, into their multivariate analysis, and no matching methods were used, so there might have been interaction effects. Second, there were little data about stratifying the impact of pre-treatment sarcopenia on survival according to clinical stages, which is commonly used for identifying higher-risk groups. For example, Van Rijn-Dekker et al. (26) found that sarcopenia is not a prognostic factor in early-stage HNSCC. Third, because Fattouh et al. (29) only reported the positive HR in their multivariate analysis, the univariate meta-analysis included 10/11 of eligible primary studies; however, according to the principle of Cox regression, there is little chance that the conclusion of the meta-analysis will be affected. Finally, the reasons for the different statistical significance between Egger's test and the trim-and-fill method might derive from the low number of included studies; however, these studies are relatively new, and we did not receive replies from the authors of three conference articles with positive (49, 50) and negative (51) results, which requires further evaluation after their official publication.

## Conclusion

The presence of pre-treatment sarcopenia has a significant negative impact on OS and RFS in HNC compared with its absence. Further well-conducted studies with detailed stratification are needed to complement our findings.

## Data Availability Statement

Please contact author for data requests.

## Author Contributions

XH, SL, and J-FL collected, extracted, and analyzed the data and wrote the paper. WW, Z-QL, and Z-JL performed quality assessment and analyzed the data. LG and H-XL conceived and designed this study. All authors reviewed the paper, read, and approved the final manuscript.

### Conflict of Interest

The authors declare that the research was conducted in the absence of any commercial or financial relationships that could be construed as a potential conflict of interest.

## Supplementary Material

Table S1 NOS score of included retrospective studies.

Table S2 Sensitivity analysis (one study was omitted in each round) of multivariate meta-analysis of OS between the sarcopenia and non-sarcopenia groups.

Figure S1 Kappa coefficient value for 38 eligible studies at full text step. Kappa value 0.0–0.20 (slight), 0.21–0.40 (fair), 0.41–0.60 (moderate), 0.61–0.80 (substantial), and 0.81–1 (almost perfect).

Figure S2 Egger's test for OS and trim-and-fill method. For Egger's test, if the test of H0: *P* > 0.1, there is no publication bias; for the trim-and-fill method, if the final conclusion from the estimated pooled HR and 95%CI remains unchanged, publication bias has little influence on the authenticity of the final conclusion.

Figure S3 Funnel plot of univariate meta-analysis for OS.

## References

[1] Noguchi M Kakuma T Uemura H Nasu Y Kumon H Hirao Y. A randomized phase II trial of personalized peptide vaccine plus low dose estramustine phosphate (EMP) versus standard dose EMP in patients with castration resistant prostate cancer. Cancer Immunol Immun. (2010) 59:1001–9. 10.1007/s00262-010-0822-4 20146063 PMC11030921

[2] Jager-Wittenaar H Dijkstra PU Vissink A Langendijk JA van der Laan BF Pruim J. Changes in nutritional status and dietary intake during and after head and neck cancer treatment. Head Neck. (2011) 33:863–70. 10.1002/hed.21546 20737491

[3] Jackson W Alexander N Schipper M Fig L Feng F Jolly S. Characterization of changes in total body composition for patients with head and neck cancer undergoing chemoradiotherapy using dual-energy x-ray absorptiometry. Head Neck. (2014) 36:1356–62. 10.1002/hed.23461 23970480

[4] Langius JA Bakker S Rietveld DH Kruizenga HM Langendijk JA Weijs PJ. Critical weight loss is a major prognostic indicator for disease-specific survival in patients with head and neck cancer receiving radiotherapy. Br J Cancer. (2013) 109:1093–9. 10.1038/bjc.2013.458 23928661 PMC3778304

[5] Pressoir M Desne S Berchery D Rossignol G Poiree B Meslier M. Prevalence, risk factors and clinical implications of malnutrition in French comprehensive cancer centres. Br J Cancer. (2010) 102:966–71. 10.1038/sj.bjc.6605578 20160725 PMC2844030

[6] Prado CM Lieffers JR McCargar LJ Reiman T Sawyer MB Martin L. Prevalence and clinical implications of sarcopenic obesity in patients with solid tumours of the respiratory and gastrointestinal tracts: a population-based study. Lancet Oncol. (2008) 9:629–35. 10.1016/S1470-2045(08)70153-0 18539529

[7] Cruz-Jentoft AJ Bahat G Bauer J Boirie Y Bruyere O Cederholm T Sarcopenia: revised European consensus on definition and diagnosis. Age Ageing. (2019) 48:16–31. 10.1093/ageing/afy169 30312372 PMC6322506

[8] Swartz JE Pothen AJ Wegner I Smid EJ Swart KM de Bree R. Feasibility of using head and neck CT imaging to assess skeletal muscle mass in head and neck cancer patients. Oral Oncol. (2016) 62:28–33. 10.1016/j.oraloncology.2016.09.006 27865369

[9] Miyamoto Y Baba Y Sakamoto Y Ohuchi M Tokunaga R Kurashige J. Sarcopenia is a negative prognostic factor after curative resection of colorectal cancer. Ann Surg Oncol. (2015) 22:2663–8. 10.1245/s10434-014-4281-6 25564158

[10] Kuroki LM Mangano M Allsworth JE Menias CO Massad LS Powell MA. Pre-operative assessment of muscle mass to predict surgical complications and prognosis in patients with endometrial cancer. Ann Surg Oncol. (2015) 22:972–9. 10.1245/s10434-014-4040-8 25190123 PMC4355998

[11] Voron T Tselikas L Pietrasz D Pigneur F Laurent A Compagnon P. Sarcopenia impacts on short- and long-term results of hepatectomy for hepatocellular carcinoma. Ann Surg. (2015) 261:1173–83. 10.1097/SLA.0000000000000743 24950264

[12] Martin L Birdsell L Macdonald N Reiman T Clandinin MT McCargar LJ. Cancer cachexia in the age of obesity: skeletal muscle depletion is a powerful prognostic factor, independent of body mass index. J Clin Oncol. (2013) 31:1539–47. 10.1200/JCO.2012.45.2722 23530101

[13] Du E Mazul AL Farquhar D Brennan P Anantharaman D Abedi-Ardekani B. Long-term survival in head and neck cancer: impact of site, stage, smoking, and human papillomavirus status. Laryngoscope. (2019) 129:2506–13. 10.1002/lary.27807 30637762 PMC6907689

[14] Giraldi L Leoncini E Pastorino R Wunsch-Filho V de Carvalho M Lopez R. Alcohol and cigarette consumption predict mortality in patients with head and neck cancer: a pooled analysis within the International Head and Neck Cancer Epidemiology (INHANCE) consortium. Ann Oncol. (2017) 28:2843–51. 10.1093/annonc/mdx486 28945835 PMC5834132

[15] Iyengar NM Kochhar A Morris PG Morris LG Zhou XK Ghossein RA. Impact of obesity on the survival of patients with early-stage squamous cell carcinoma of the oral tongue. Cancer. (2014) 120:983–91. 10.1002/cncr.28532 24449483 PMC3961521

[16] Rietbergen MM Brakenhoff RH Bloemena E Witte BI Snijders PJ Heideman DA. Human papillomavirus detection and comorbidity: critical issues in selection of patients with oropharyngeal cancer for treatment De-escalation trials. Ann Oncol. (2013) 24:2740–5. 10.1093/annonc/mdt319 23946330

[17] Cho Y Kim JW Keum KC Lee CG Jeung HC Lee IJ. Prognostic significance of sarcopenia with inflammation in patients with head and neck cancer who underwent definitive chemoradiotherapy. Front Oncol. (2018) 8:457. 10.3389/fonc.2018.00457 30460194 PMC6232888

[18] Grossberg AJ Chamchod S Fuller CD Mohamed AS Heukelom J Eichelberger H. Association of body composition with survival and locoregional control of radiotherapy-treated head and neck squamous cell carcinoma. JAMA Oncol. (2016) 2:782–9. 10.1001/jamaoncol.2015.6339 26891703 PMC5080910

[19] Talwar B Donnelly R Skelly R Donaldson M. Nutritional management in head and neck cancer: United Kingdom National Multidisciplinary Guidelines. J Laryngol Otol. (2016) 130:S32–40. 10.1017/S0022215116000402 27841109 PMC4873913

[20] Ganju RG Morse R Hoover A TenNapel M Lominska CE. The impact of sarcopenia on tolerance of radiation and outcome in patients with head and neck cancer receiving chemoradiation. Radiother Oncol. (2019) 137:117–24. 10.1016/j.radonc.2019.04.023 31085391

[21] Stone L Olson B Mowery A Krasnow S Jiang A Li R. Association between sarcopenia and mortality in patients undergoing surgical excision of head and neck cancer. JAMA Otolaryngol Head Neck Surg. (2019) 145:647–54. 10.1001/jamaoto.2019.1185 31169874 PMC6555480

[22] Bril SI Pezier TF Tijink BM Janssen LM Braunius WW de Bree R. Preoperative low skeletal muscle mass as a risk factor for pharyngocutaneous fistula and decreased overall survival in patients undergoing total laryngectomy. Head Neck. (2019) 41:1745–55. 10.1002/hed.25638 30663159 PMC6590286

[23] Wendrich AW Swartz JE Bril SI Wegner I de Graeff A Smid EJ. Low skeletal muscle mass is a predictive factor for chemotherapy dose-limiting toxicity in patients with locally advanced head and neck cancer. Oral oncol. (2017) 71:26–33. 10.1016/j.oraloncology.2017.05.012 28688687

[24] Jung AR Roh JL Kim JS Kim SB Choi SH Nam SY. Prognostic value of body composition on recurrence and survival of advanced-stage head and neck cancer. Eur J Cancer. (2019) 116:98–106. 10.1016/j.ejca.2019.05.006 31185387

[25] Mourtzakis M Prado CM Lieffers JR Reiman T McCargar LJ Baracos VE. A practical and precise approach to quantification of body composition in cancer patients using computed tomography images acquired during routine care. Appl Physiol Nutr Metab. (2008) 33:997–1006. 10.1139/H08-075 18923576

[26] Van Rijn-Dekker I Van den Bosch L Van den Hoek A Bijl H Dieters M Van Aken E Impact of sarcopenia on survival and late toxicity in head and neck cancer patients treated with RT. Radiother Oncol. (2019) 133:S197–8. 10.1016/S0167-8140(19)30813-8 32251949

[27] Go SI Park MJ Song HN Kang MH Park HJ Jeon KN. Sarcopenia and inflammation are independent predictors of survival in male patients newly diagnosed with small cell lung cancer. Support Care Cancer. (2016) 24:2075–84. 10.1007/s00520-015-2997-x 26546456

[28] Kim YS Lee Y Chung YS Lee DJ Joo NS Hong D. Prevalence of sarcopenia and sarcopenic obesity in the Korean population based on the Fourth Korean National Health and Nutritional Examination Surveys. J Gerontol A Biol Sci Med Sci. (2012) 67:1107–13. 10.1093/gerona/gls071 22431554

[29] Fattouh M Chang GY Ow TJ Shifteh K Rosenblatt G Patel VM. Association between pretreatment obesity, sarcopenia, and survival in patients with head and neck cancer. Head Neck. (2018) 41:707–14. 10.1002/hed.25420 30582237 PMC6709588

[30] Prado CM Baracos VE McCargar LJ Reiman T Mourtzakis M Tonkin K. Sarcopenia as a determinant of chemotherapy toxicity and time to tumor progression in metastatic breast cancer patients receiving capecitabine treatment. Clin Cancer Res. (2009) 15:2920–6. 10.1158/1078-0432.CCR-08-2242 19351764

[31] Mokdad AH Ford ES Bowman BA Dietz WH Vinicor F Bales VS. Prevalence of obesity, diabetes, and obesity-related health risk factors, 2001. JAMA. (2003) 289:76–9. 10.1001/jama.289.1.76 12503980

[32] Parsons HA Baracos VE Dhillon N Hong DS Kurzrock R. Body composition, symptoms, and survival in advanced cancer patients referred to a phase I service. PLoS ONE. (2012) 7:e29330. 10.1371/journal.pone.0029330 22235285 PMC3250428

[33] Nishikawa D Hanai N Suzuki H Koide Y Beppu S Hasegawa Y. The impact of skeletal muscle depletion on head and neck squamous cell carcinoma. ORL. (2018) 80:1–9. 10.1159/000485515 29393251

[34] Tamaki A Manzoor NF Babajanian E Ascha M Rezaee R Zender CA. Clinical significance of sarcopenia among patients with advanced oropharyngeal cancer. Otolaryngol Head Neck Surg. (2018) 160:480–7. 10.1177/0194599818793857 30105922

[35] Wells G Shea BJ O'Connell D Peterson J Welch V Losos M The Newcastle-Ottawa Scale (NOS) for assessing the quality of nonrandomised studies in meta-analyses. Available online at: http://www.ohri.ca/programs/clinical_epidemiology/oxford.asp

[36] Parmar MK Torri V Stewart L. Extracting summary statistics to perform meta-analyses of the published literature for survival endpoints. Stat Med. (1998) 17:2815–34. 10.1002/(sici)1097-0258(19981230)17:24<2815::aid-sim110>3.0.co;2-8 9921604

[37] Tierney JF Stewart LA Ghersi D Burdett S Sydes MR. Practical methods for incorporating summary time-to-event data into meta-analysis. Trials. (2007) 8:16. 10.1186/1745-6215-8-16 17555582 PMC1920534

[38] Egger M Davey Smith G Schneider M Minder C. Bias in meta-analysis detected by a simple, graphical test. BMJ. (1997) 315:629–34. 10.1136/bmj.315.7109.629 9310563 PMC2127453

[39] Duval S Tweedie R. Trim and fill: a simple funnel-plot-based method of testing and adjusting for publication bias in meta-analysis. Biometrics. (2000) 56:455–63. 10.1111/j.0006-341X.2000.00455.x 10877304

[40] Shachar SS Williams GR Muss HB Nishijima TF. Prognostic value of sarcopenia in adults with solid tumours: A meta-analysis and systematic review. Eur J Cancer. (2016) 57:58–67. 10.1016/j.ejca.2015.12.030 26882087

[41] Chang KV Chen JD Wu WT Huang KC Hsu CT Han DS. Association between loss of skeletal muscle mass and mortality and tumor recurrence in hepatocellular carcinoma: a systematic review and meta-analysis. Liver Cancer. (2018) 7:90–103. 10.1159/000484950 29662836 PMC5892377

[42] Gorenc M Kozjek NR Strojan P. Malnutrition and cachexia in patients with head and neck cancer treated with (chemo) radiotherapy. Rep Pract Oncol Radiother. (2015) 20:249–58. 10.1016/j.rpor.2015.03.001 26109912 PMC4477124

[43] Achim V Bash J Mowery A Guimaraes AR Li R Schindler J. prognostic indication of sarcopenia for wound complication after total laryngectomy. JAMA Otolaryngol Head Neck Surg. (2017) 143:1159. 10.1001/jamaoto.2017.0547 28448668

[44] Ozola Zalite I Zykus R Francisco Gonzalez M Saygili F Pukitis A Gaujoux S. Influence of cachexia and sarcopenia on survival in pancreatic ductal adenocarcinoma: a systematic review. Pancreatology. (2015) 15:19–24. 10.1016/j.pan.2014.11.006 25524484

[45] Ganju RG Morse R Tennapel MJ Hoover A Kakarala K Shnayder L Skeletal muscle gauge measured at the c3 vertebral body predicts for outcomes in men with P16-positive oropharynx cancer. Int J Radiat Oncol Biol Phys. (2019) 105:E420 10.1016/j.ijrobp.2019.06.1528

[46] Freiberger E Sieber C Pfeifer K. Physical activity, exercise, and sarcopenia – future challenges. Wien Med Wochenschr. (2011) 161:416–25. 10.1007/s10354-011-0001-z 21792532

[47] Morley JE Argiles JM Evans WJ Bhasin S Cella D Deutz NE. Nutritional recommendations for the management of sarcopenia. J Am Med Dir Assoc. (2010) 11:391–6. 10.1016/j.jamda.2010.04.014 20627179 PMC4623318

[48] Cohen S Nathan JA Goldberg AL. Muscle wasting in disease: molecular mechanisms and promising therapies. Nat Rev Drug Discov. (2015) 14:58–74. 10.1038/nrd4467 25549588

[49] Chamchod S Fuller CD Grossberg AJ Mohamed AS Heukelom J ichelberger H sarcopenia/cachexia is associated with reduced survival and locoregional control in head and neck cancer patients receiving radiotherapy: results from quantitative imaging analysis of lean body mass. Oncology. (2015) 29(4 Suppl 1):205153. 25930908

[50] Inokuchi H Okano K Takehana K Tsutsui K Hiraoka M Prognostic impact of quantitative imaging analysis of lean body mass after chemoradiation therapy for patients with advanced nasopharyngeal cancer. Int J Radiat Oncol Biol Phys. (2018) 100:1342 10.1016/j.ijrobp.2017.12.094

[51] Kabarriti R Ohri N Bontempo A Romano M Modi C Viswanathan S The impact of dietary regimen compliance and sarcopenia in head and neck cancer patients treated with definitive radiation therapy. Int J Radiat Oncol Biol Phys. (2015) 93:E332–3. 10.1016/j.ijrobp.2015.07.1395

