# Supplementary material for: When the Loss Costs Too Much: A Systematic Review and Meta-Analysis of Sarcopenia in Head and Neck Cancer
Source: Front Oncol. 2020 Feb 5;9:1561. doi: 10.3389/fonc.2019.01561 (PMC7012991; doi:10.3389/fonc.2019.01561)
Supplement: Table S1 — NOS score of included retrospective studies. [file Table_1.docx]

**Table S1. NOS score of included retrospective studies.**

|  |  |  |  |  |  |  |  |  |  |
| --- | --- | --- | --- | --- | --- | --- | --- | --- | --- |
| **Cohorts** | **Representativeness of the exposed cohort** | **Selection of the non-exposed cohort** | **Ascertainment of exposure** | **Demonstration that outcome of interests was not present at start of study** | **Comparability of cohorts on the basis of the design or analysis** | **Assessment of outcocme** | **Was follow-up long enough for outcome to occur** | **Adequacy of follow up of cohorts** | **Quality score** |
| Stone et al,2019 | ★ | ★ | ★ | ★ | ★★ | ★ | ☆ | ☆ | **7** |
| Jung et al,2019 | ☆ | ★ | ★ | ★ | ★★ | ★ | ★ | ☆ | **7** |
| Ganju et al, 2019 | ★ | ★ | ★ | ★ | ★★ | ★ | ☆ | ☆ | **7** |
| Bril et al, 2019 | ★ | ★ | ★ | ★ | ★☆ | ★ | ★ | ☆ | **7** |
| Van Rijn - Dekker et al 2019 | ★ | ★ | ★ | ★ | ★☆ | ★ | ☆ | ☆ | **6** |
| Cho et al，2018 | ★ | ★ | ★ | ★ | ★★ | ★ | ☆ | ☆ | **7** |
| Nishikawa et al, 2018 | ★ | ★ | ★ | ★ | ★☆ | ★ | ☆ | ☆ | **6** |
| Tamaki et al, 2018 | ☆ | ★ | ★ | ★ | ★★ | ★ | ☆ | ☆ | **6** |
| *Fattouh et al, 2018 | ★ | ★ | ★ | ★ | ★★ | ★☆ | ★ | ☆ | **8** |
| Wendrichet al, 2017 | ★ | ★ | ★ | ☆ | ★☆ | ★ | ★ | ☆ | **6** |
| Grossberg et al, 2016 | ★ | ★ | ★ | ★ | ★★ | ★ | ★ | ☆ | **8** |

* nested case-control

**TableS2. Sensitivity analysis (one study was omitted in each round) ofmultivariate meta-analysis of OS between the sarcopenia and non-sarcopenia groups.**

| **One omitted study** | **HR (95%CI) remained** | **Heterogeneity remained** | |
| --- | --- | --- | --- |
|  |  | **I^2^ (%)** | **P value** |
| Grossberg 2016 | 2.22 [1.65, 2.99] | 57 | 0.02 |
| Nishikawa 2018 | 2.10 [1.61, 2.74] | 53 | 0.04 |
| Tamaki 2018 | 2.20 [1.65, 2.93] | 57 | 0.02 |
| Fattouh 2018 | 2.09 [1.59, 2.75] | 52 | 0.04 |
| Stone 2019 | 2.13 [1.61, 2.81] | 55 | 0.03 |
| **Van Rijn - Dekker 2019** | **2.34 [1.88, 2.92]** | **6** | **0.38** |
| Ganju 2019 | 2.20 [1.64, 2.94] | 57 | 0.02 |
| **Jung 2019** | **1.79 [1.49, 2.15]** | **6** | **0.38** |
| Bril 2019 | 2.24 [1.65, 3.05] | 56 | 0.02 |
