# Supplementary figures and images for: When the Loss Costs Too Much: A Systematic Review and Meta-Analysis of Sarcopenia in Head and Neck Cancer
Source: Front Oncol. 2020 Feb 5;9:1561. doi: 10.3389/fonc.2019.01561 (PMC7012991; doi:10.3389/fonc.2019.01561)

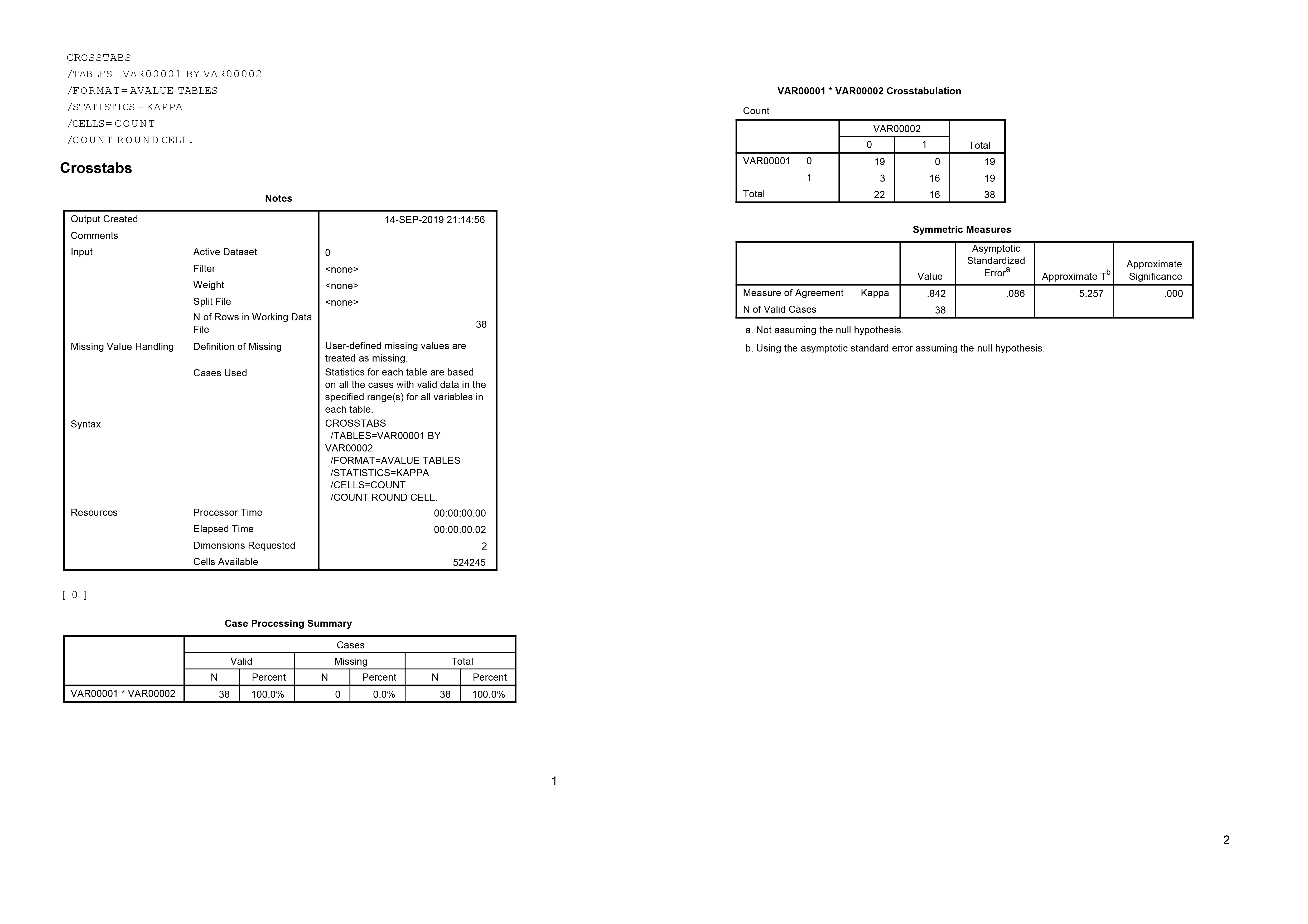

Supplement: Figure S1 — Kappa coefficient value for 38 eligible studies at full text step. Kappa value 0.0–0.20 (slight), 0.21–0.40 (fair), 0.41–0.60 (moderate), 0.61–0.80 (substantial), and 0.81–1 (almost perfect). [file Image_1.TIF]

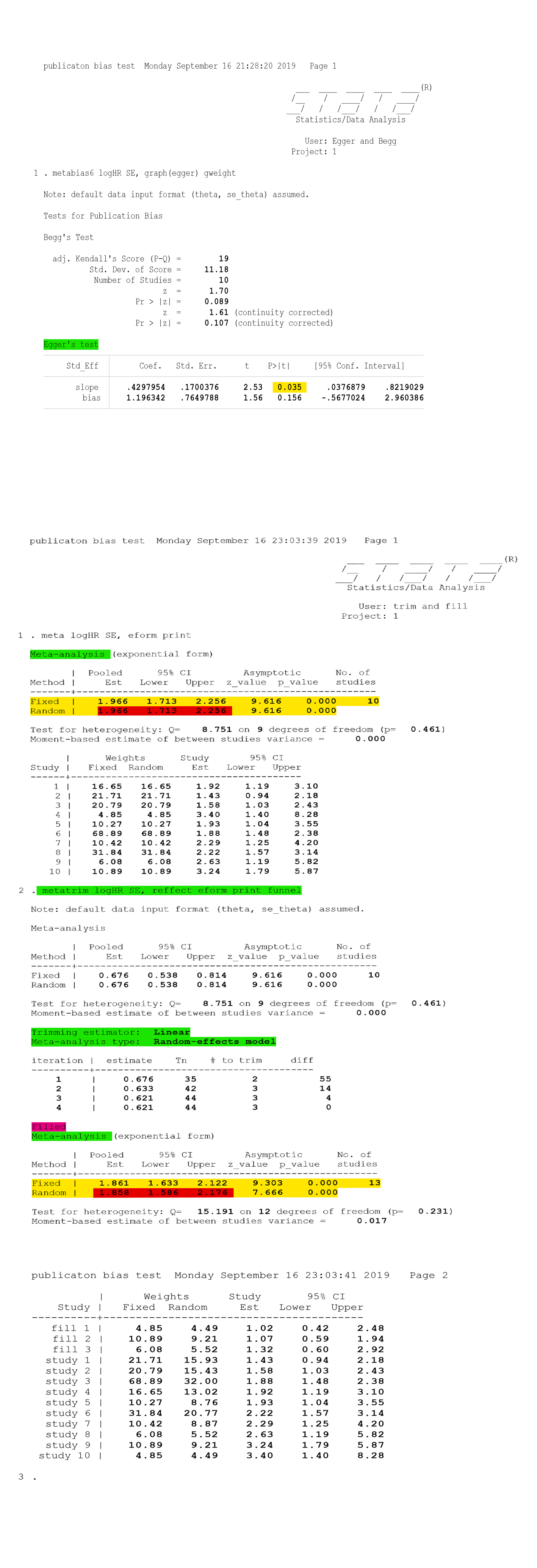

Supplement: Figure S2 — Egger's test for OS and trim-and-fill method. For Egger's test, if the test of H0: P > 0.1, there is no publication bias; for the trim-and-fill method, if the final conclusion from the estimated pooled HR and 95%CI remains unchanged, publication bias has little influence on the authenticity of the final conclusion. [file Image_2.TIF]

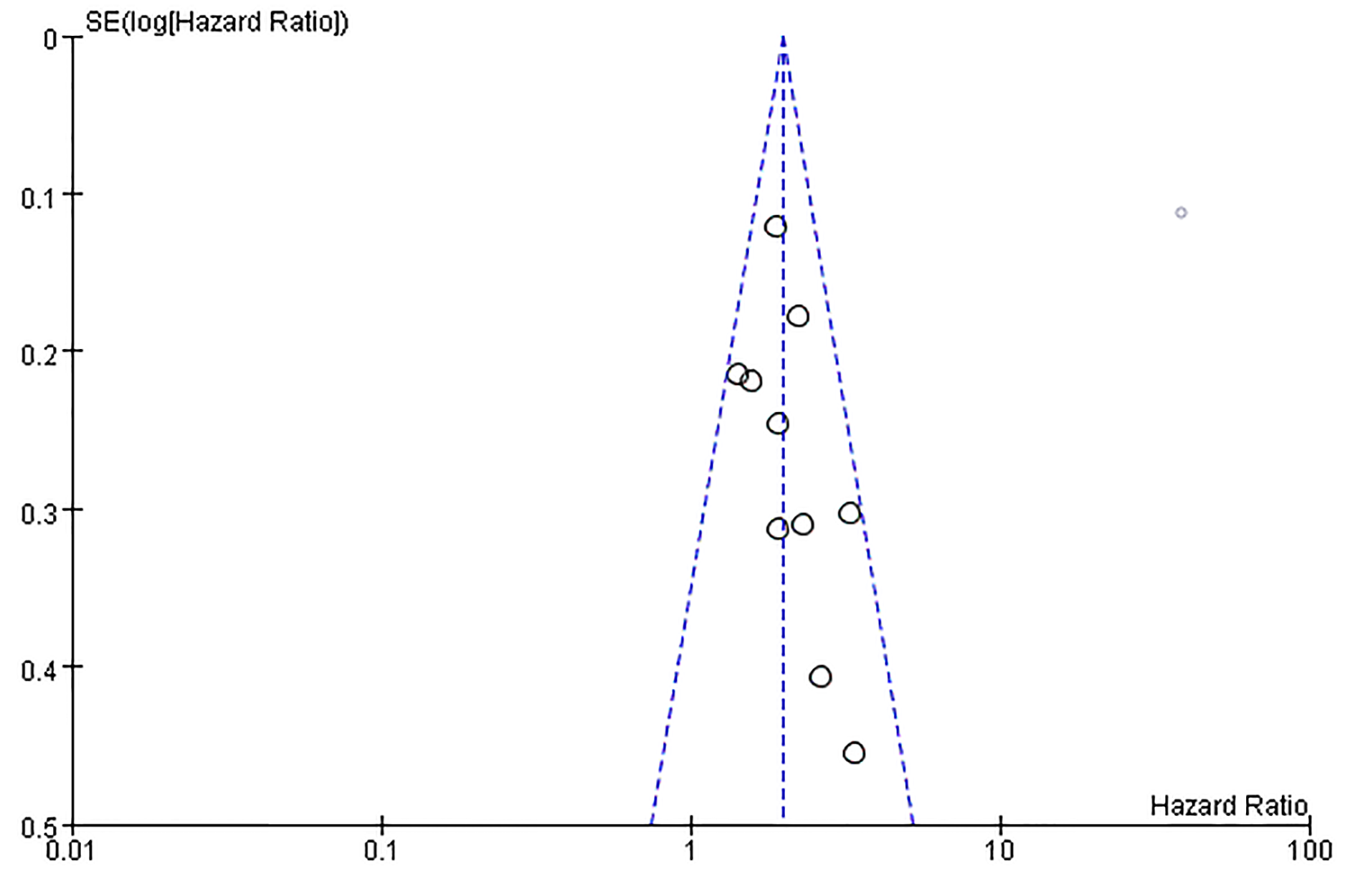

Supplement: Figure S3 — Funnel plot of univariate meta-analysis for OS. [file Image_3.TIF]
